# Susceptibility of *Anopheles gambiae* to Natural *Plasmodium falciparum* Infection: A Comparison between the Well-Established *Anopheles gambiae* s.s Line and a Newly Established Ugandan *Anopheles gambiae* s.s. Line

**DOI:** 10.4269/ajtmh.23-0203

**Published:** 2023-12-26

**Authors:** Daniel Ayo, Ismail Onyige, Joseph Okoth, Eric Musasizi, Ambrose Oruni, Jordache Ramjith, Emmanuel Arinaitwe, John C. Rek, Chris Drakeley, Sarah G. Staedke, Martin J. Donnelly, Teun Bousema, Melissa Conrad, Sara Lynn Blanken

**Affiliations:** ^1^ Infectious Diseases Research Collaboration, Nagongera Hospital, Tororo, Uganda;; ^2^ Department of Vector Biology, Liverpool School of Tropical Medicine, Liverpool, United Kingdom;; ^3^ Department for Health Evidence, Radboud University Medical Center, Nijmegen, Netherlands;; ^4^ Department of Infection Biology, London School of Hygiene and Tropical Medicine, London, United Kingdom;; ^5^ Department of Medical Microbiology, Radboud University Nijmegen Medical Center, Nijmegen;; ^6^ Department of Medicine, San Francisco General Hospital, University of California, San Francisco, California

## Abstract

Much of our understanding of malaria transmission comes from mosquito feeding assays using *Anopheles* mosquitoes from colonies that are well adapted to membrane feeding. This raises the question whether results from colony mosquitoes lead to overestimates of outcomes in wild *Anopheles* mosquitoes. We successfully established an *Anopheles* colony using progeny of wild *Anopheles gambiae* s.s. mosquitoes (Busia mosquitoes) and directly compared their susceptibility to infection with *Plasmodium falciparum* with the widely used *An. gambiae* s.s. mosquitoes (Kisumu mosquitoes) using gametocyte-infected Ugandan donor blood. The proportion of infectious feeds did not differ between Busia (71.8%, 23/32) and Kisumu (68.8%, 22/32, *P* = 1.00) mosquitoes. When correcting for random effects of donor blood, we observed a 23% higher proportion of infected Busia mosquitoes than infected Kisumu mosquitoes (RR, 1.23; 95% CI, 1.10–1.38, *P <* 0.001). This study suggests that feeding assays with Kisumu mosquitoes do not overestimate outcomes in wild *An. gambiae* s.s. mosquitoes, the mosquito species most relevant to malaria transmission in Uganda.

Malaria remains a global health problem despite ongoing control efforts.^1^^,^^2^ Understanding malaria transmission is essential for the development and deployment of effective interventions to reduce transmission. Human-to-mosquito malaria transmission is mediated by sexual stage parasites (gametocytes) taken up by female mosquito vectors during a blood meal.

Several dozens of *Anopheles* species transmit malaria with variable vectorial capacity.^3^ *Anopheles* mosquito species can differ in behavior, immunity, microbiota, and susceptibility to *Plasmodium* parasites.^3^^–^^7^ The abundance of transmission-competent vectors differs by region. In Uganda, *Anopheles gambiae* sensu lato (s.l.) and *Anopheles funestus* s.l. are the dominant vector groups.^4^ *Anopheles* complex species are further divided into genetically distinct but morphologically identical subspecies. In Uganda, *An. gambiae* sensu stricto (s.s) dominates and differs from other *An*. *gambiae* subspecies, such as *An. arabiensis*, in biting behavior and host preference.^4^

Much of our understanding of malaria transmission comes from mosquito feeding assays using *Anopheles* mosquitoes that have been maintained in a laboratory colony for many, sometimes hundreds of, generations.^8^^–^^10^ During a feeding experiment, mosquitoes are typically fed on venous blood through artificial membranes. Colony mosquitoes are selected for culture adaptability, aggressivity, and permissiveness.^11^ Optimizing feeding conditions under laboratory settings can increase mosquito body size,^12^ ingested blood volume, and susceptibility to *Plasmodium* infection.^13^ This raises the question whether feeding assays using long-time laboratory-adapted *Anopheles* mosquitoes may lead to overestimates of outcomes observed with more natural (wild or recently colony-adapted) *Anopheles* mosquitoes.

Here, we studied susceptibility of *An. gambiae* mosquitoes to *Plasmodium falciparum* infection using two mosquito sources. We compared infection rates between recently colonized mosquitoes from Uganda and mosquitoes from a widely used *An. gambiae* s.s. colony from Kenya established in 1975.^14^

*Anopheles gambiae* s.l. mosquitoes were collected from Busia, an area in eastern Uganda characterized by high mosquito densities and intense malaria transmission with limited control measures.^15^ Indoor resting blood-fed mosquitoes were collected using an electric aspirator in November 2018 to establish progeny broods at the Liverpool School of Tropical Medicine (LSTM), United Kingdom (A. Oruni et al., unpublished data). During establishment of the Busia colony, field collections were initially mixed, with a small proportion of *An. arabiensis* lost in the first generation, resulting in an *An. gambiae* s.s. second-generation population verified by species diagnostic polymerase chain reaction.^16^ After establishment at LSTM, the Busia colony was transferred to an insectary in Butabika, Uganda, in October 2019. In January 2020, the colony was transferred to Nagongera, where the present study was conducted. Insectary conditions were identical throughout this process (±2–25°C; ±10–80 mm Hg).

The mosquitoes from Busia (Busia mosquitoes) were reared alongside an established *An. gambiae* s.s. Kisumu colony (Kisumu mosquitoes) in independent, adjacent insectaries but under identical conditions, including temperature and relative humidity (±2–25°C, ±10–80 mm Hg, respectively).^17^ Larvae (L1-L2) were fed with Liquifry (NO1, Interpet, UK) or Cichlid (King British, UK)) and reared at 30–35°C until pupation. After hatching, adult mosquitoes were maintained on 10% glucose solution. Colony maintenance relied on malaria-free fresh blood and 10% glucose solution. Using these procedures, we were able to weekly produce ∼500 female Busia and ∼1,150 female Kisumu mosquitoes. Mosquito wing size was microscopically measured for body size estimation, using a micrometer ocular, with measurements from the alula to the end of R2 vein (Supplemental Figure 1).

Gametocyte donors were selected from clinical patients at Tororo General Hospital. Thick blood smears were stained with 10% Giemsa and microscopically examined at 100× magnification for the presence of gametocytes. Gametocytes were counted against 500 white blood cells using 100 microscopy fields.Gametocyte-positive individuals that had not received antimalarial treatment since the onset of symptoms were invited to donate blood for membrane feeding experiments. Gametocyte density was not molecularly determined, as the primary comparison focused on different mosquito sources feeding on the same blood material. Approximately 4 mL of donor blood was collected in lithium heparin tubes for use in feeding assays.^18^ Using a prewarmed water jacket glass feeder system, 3- to 5-day-old females of Busia (50–80 per experiment) and Kisumu (51–81 per experiment) mosquitoes were fed on blood from the same donor (Supplemental Figure 2). When volume allowed, an ∼6-fold-gametocyte-enriched blood meal was obtained using magnetic cell sorting (MACS)^19^ and offered to both mosquito sources. Mosquitoes were allowed to feed for exactly 30 minutes; unfed mosquitoes were recorded and removed. Mosquitoes were maintained at the insectary and dissected 10 days after feeding in 1% mercurochrome. The presence of oocysts was examined microscopically at 40× magnification.

A Wilcoxon rank sum test was used to compare median wing sizes of Busia and Kisumu mosquitoes, the proportions of infected Busia and Kisumu mosquitoes, and the average numbers of oocysts per infected Busia or Kisumu mosquito (oocyst density). In this way, one individual contributed one mean oocyst density observation per mosquito source and feed type. A mixed-effects binomial regression with a log link for proportion of infected mosquitoes and a negative binomial link for mean oocyst density was used while accounting for correlations between feeding outcomes on the same donor blood using random effects. A two-proportion *z*-test was used to compare infected Busia and Kisumu mosquitoes within the total dissected. Statistical analyses were performed using R (v. 2022.02.1). A *P* value of 0.05 or less was considered significant.

Written informed consent was received from all eligible study participants. This study was approved by the Makerere University Research and Ethics Committee, the Uganda National Council for Science and Technology, and the University of California, San Francisco, Committee on Human Research.

In January 2022 and March 2022, mosquito wing size was measured for a total of 70 Busia and 80 Kisumu mosquitoes (Supplemental Figure 1). Wing size measurements took place more than 3 years after the colony was first established, thus not capturing changes in mosquito size during colonization. Throughout our study period, median wing size did not change over time for Busia (*P* = 0.253) or Kisumu (*P* = 0.297) mosquitoes. The median wing size of Busia mosquitoes (2.80 mm; interquartile range [IQR], 2.68–2.88 mm) was significantly smaller than the wing size of Kisumu mosquitoes (2.88 mm; IQR, 2.80–2.96 mm, *P* = 0.0002) (Figure 1A).

**Figure 1.**
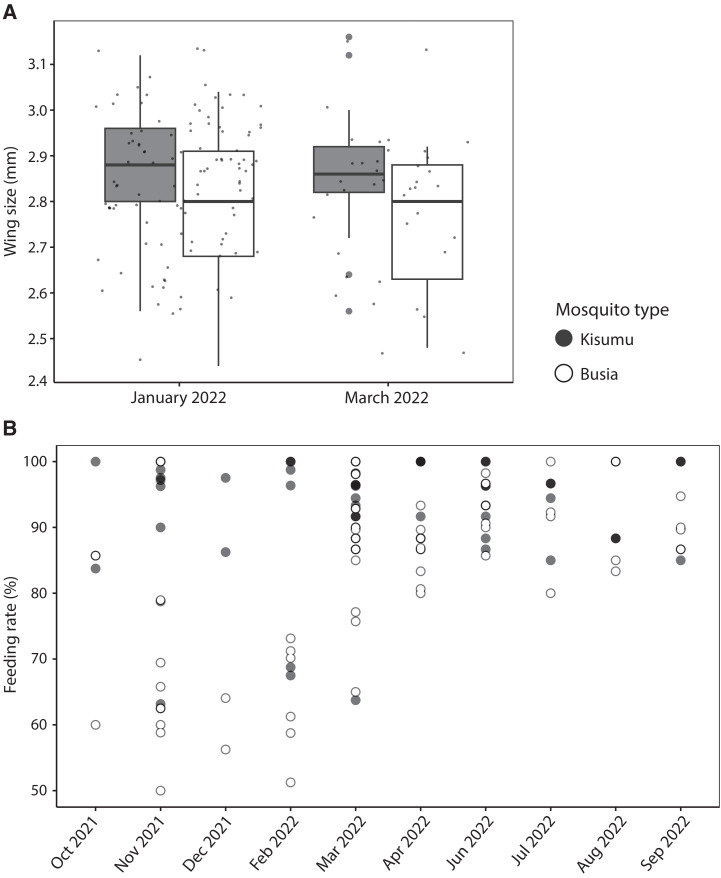
Comparison of wing sizes (**A**) and feeding rates in membrane feeding assays (**B**) using newly established Busia and well-established Kisumu mosquitoes.

We conducted 134 feeding assays on 55 donors positive for gametocytes by microscopy. From these, 22 donors infected at least one mosquito from either mosquito source. Of these, 11 donors provided enough blood to conduct both a MACS-concentrated and a physiological gametocyte concentration feed, resulting in 22 paired feeds that allowed comparisons between different gametocyte concentrations. Another four and six donors participated in only a physiological or a MACS-enriched gametocyte concentration feed, respectively. By combining feeds with physiological (*n =* 15) and MACS-enriched (*n =* 17) gametocyte concentrations, we achieved 32 paired feeding assays that allowed comparisons between mosquito sources. The number of fully fed Kisumu mosquitoes was significantly higher (*P <* 0.0001) than that of Busia mosquitoes under the same laboratory and physiological conditions, highlighting the relative advantage of the established Kisumu colony of *Anopheles* mosquitoes in terms of feeding efficiencies (Figure 1B). In line with expectations,^19^ mosquitoes fed on gametocyte-enriched blood resulted in a higher proportion of infected mosquitoes than did mosquitoes fed on blood with a physiological gametocyte concentration (RR, 1.54; 95% CI, 1.26–1.89, *P <* 0.001). Our study was not designed to assess mosquito infection rates in relation to gametocyte densities but allowed direct comparisons between infection rates in the two mosquito sources feeding on the same blood material. The proportion of feeds with at least one infected mosquito did not differ significantly between Busia (71.8%, 23/32) and Kisumu (68.8%, 22/32, *P* = 1.00) mosquitoes. Among feeds with at least one infected mosquito, the median proportion of infected mosquitoes did not differ significantly between Busia (0.23; IQR, 0.04–0.39) and Kisumu (0.11; IQR, 0.03–0.27, *P* = 0.271) mosquitoes (Figure 2A and 2C). When using a more elaborate statistical approach that includes a random donor effect, we observed a 23% higher proportion of infected mosquitoes in the Busia mosquitoes than in the Kisumu colony mosquitoes (RR, 1.23; 95% CI, 1.10–1.38, *P <* 0.001). The mean oocyst density of feeds with at least one infected mosquito did not differ significantly between Busia and Kisumu mosquitoes (*P* = 0.885, Figure 2B). When applying a regression model incorporating random donor effects, we observed a 12% higher mean oocyst density in Busia mosquitoes than in Kisumu mosquitoes (RR, 1.12; 95% CI, 1.12–1.13, *P <* 0.001). Mosquito source had no significant influence (*P* = 0.409) on the relationship between mean oocyst density and oocyst prevalence (Figure 2D). These results demonstrate that both Busia and Kisumu mosquitoes were susceptible to natural *P. falciparum* infection, with some evidence for higher receptivity of the recently colonized Busia mosquito population.

**Figure 2.**
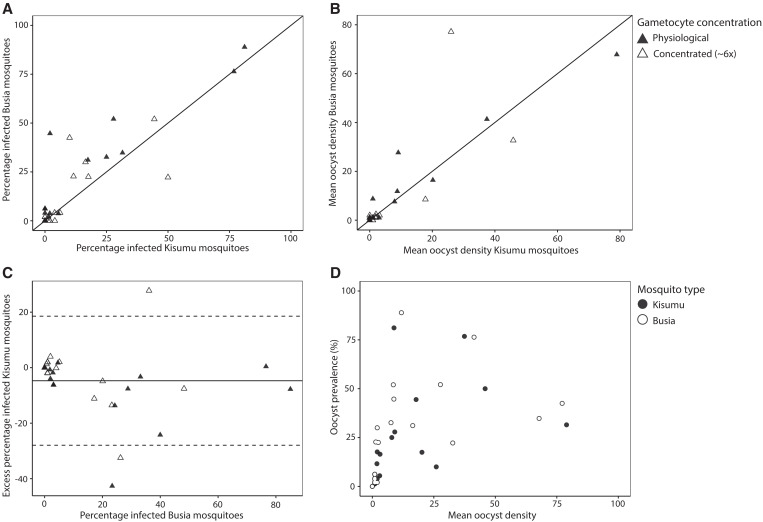
Comparison of susceptibilities of newly established Busia and well-established Kisumu *An. gambiae* mosquitoes to *P. falciparum* infection in membrane feeding assays. (**A**) The proportion of infected Busia mosquitoes (*y*-axis) is plotted against that of Kisumu mosquitoes (*x*-axis) for 32 paired feeding assays. The solid line is the line of perfect agreement. For panels **A** to **C**, solid triangles indicate that feeding was performed with physiological gametocyte concentrations, and open triangles indicate that feeding was performed with concentrated gametocytes. (**B**) The mean oocyst density of fully fed Busia mosquitoes (*y*-axis) is plotted against that of Kisumu mosquitoes (*x*-axis). The solid line is the line of perfect agreement. (**C**) The excess proportion of infected Kisumu mosquitoes (*y*-axis) is plotted against the mean percentage of infected Busia mosquitoes (*x*-axis). The limits of agreement are indicated as the mean difference (middle solid line), and the 95% CI of the limit of agreement (mean ± 1.96 SD of differences) are indicated with horizontal dotted lines. (**D**) The oocyst prevalence is plotted as the mean oocyst density for Busia (open circles) and Kisumu (solid circles) mosquitoes.

The present study indicates that well-established Kisumu colony *An. gambiae* s.s. mosquitoes do not exhibit an increased susceptibility to *P. falciparum* infection compared with that of recently adapted mosquitoes; we even observed a small but significant increase in infection rates with recently adapted Busia mosquitoes, although the comparison was restricted to microscopy-positive gametocyte carriers. Busia mosquitoes were significantly smaller and had lower feeding efficiency than Kisumu colony mosquitoes. Together, our findings suggest that outcomes from membrane feeding assays with colony *An. gambiae* s.s. mosquitoes are likely generalizable to outcomes in wild *An. gambiae* s.s. mosquitoes, the most relevant mosquito species transmitting malaria in Uganda.

## Supplemental files

10.4269/ajtmh.23-0203 Supplemental Materials
