# Supplementary material for: Susceptibility of Anopheles gambiae to Natural Plasmodium falciparum Infection: A Comparison between the Well-Established Anopheles gambiae s.s Line and a Newly Established Ugandan Anopheles gambiae s.s. Line
Source: Am J Trop Med Hyg. 2023 Dec 26;110(2):209–13. doi: 10.4269/ajtmh.23-0203 (PMC10859803; doi:10.4269/ajtmh.23-0203)

Figure S1. Mosquito wing size was measured from the alula to the end of the R2 vein microscopically using an ocular with ruler.

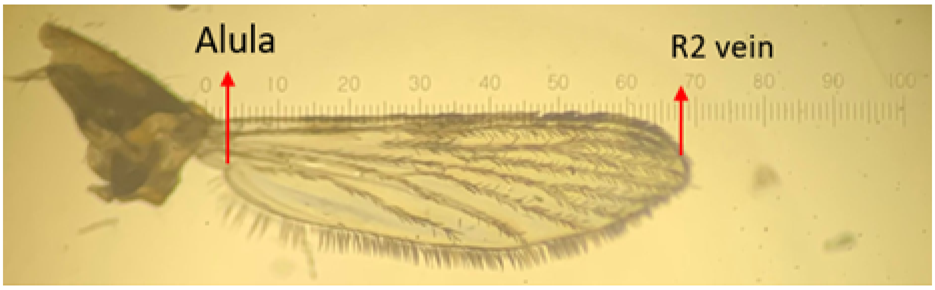

Figure S2. Comparison of the number of recently-established Busia versus long-established Kisumu mosquitoes allowed to feed over time.

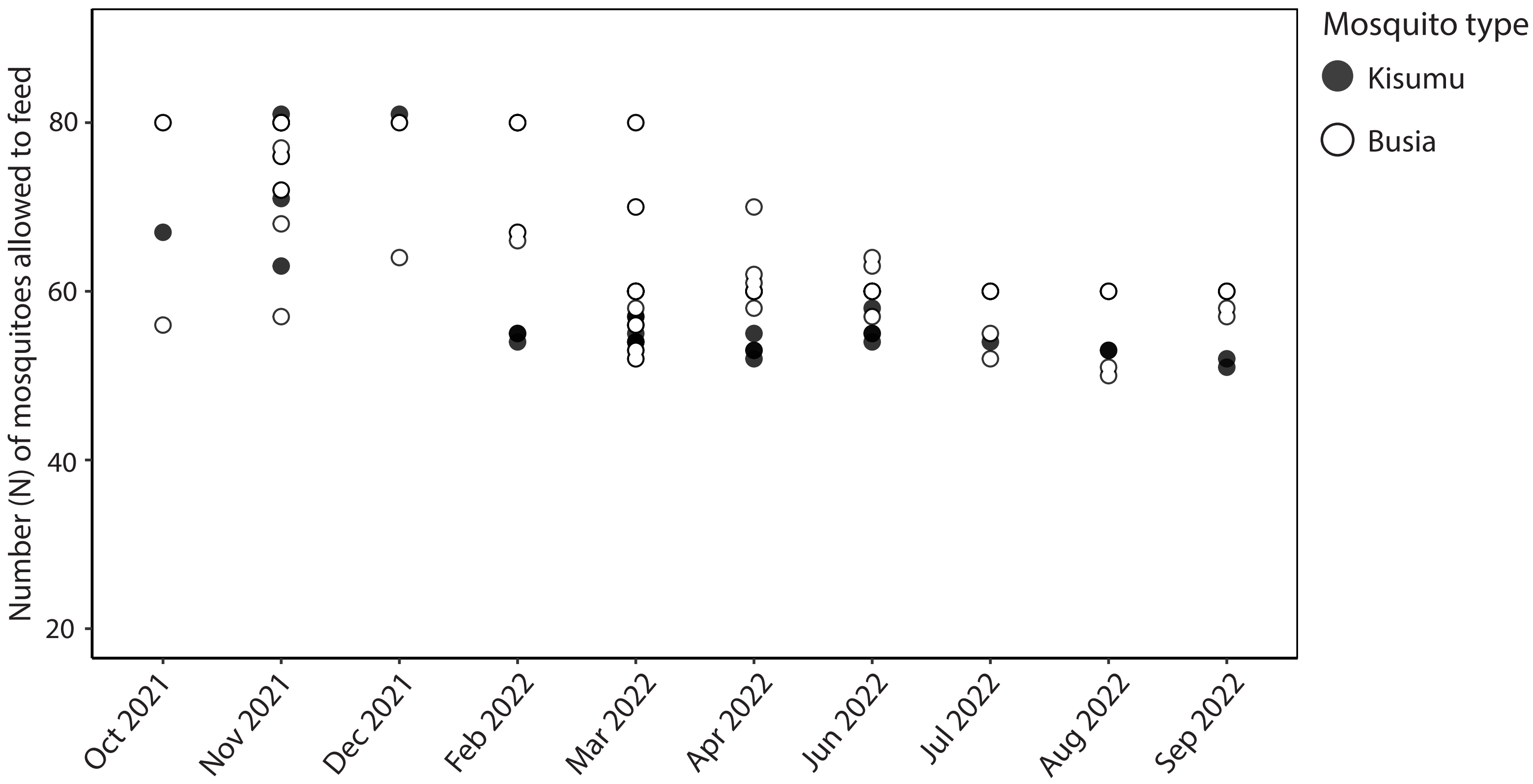

Supplement: Supplemental Materials [file tpmd230203.SD1.pdf]
